# Ejaculated boar spermatozoa displaying a rare multivesicular defect

**DOI:** 10.1186/s13028-018-0375-7

**Published:** 2018-03-27

**Authors:** Szabolcs Nagy, Tuire Tamminen, Magnus Andersson, Heriberto Rodriguez-Martinez

**Affiliations:** 1 Department of Animal Sciences, Georgikon Faculty, University of Pannonia, Deak F. u. 16, Keszthely, 8360 Hungary; 2 Saari Unit, Department of Production Animal Medicine, Faculty of Veterinary Medicine, University of Helsinki, Pohjoinen pikatie 800, 04920 Saarentaus, Finland; 3 Department of Clinical and Experimental Medicine-BKH/O&G, Linköping University, 581 85 Linköping, Sweden

**Keywords:** CLSM, Exosomes, Pig, Semen morphology, TEM

## Abstract

Two cases of a previously unreported sperm defect appearing in boar studs in Finland are presented. Spermatozoa showed small particles scattered on their surface with a prevalence decreasing with boar age. Semen samples, either stained with eosin-nigrosin or examined with phase contrast optics on formaldehyde-fixed spermatozoa, revealed the presence of multiple particles attached to the surface of spermatozoa counted as dead cells at fixation. Transmission electron microscopy revealed these were multivesicular and multilamellar vesicles, built up by phospholipid membranes. The case is classified as a post-epididymal multivesicular sperm defect with a favorable prognosis.

## Findings

In order to be able to produce acceptable ejaculates, both spermatogenesis and hormonal production and the process of sperm maturation during epididymal transit should be normal [1]. Assessment of sperm morphology is a fundamental component of the routine evaluation of semen quality. Sperm morphology reveals testicular, epididymal and even accessory gland dysfunctions that can impair, directly or indirectly, the fertilizing capacity of the ejaculated spermatozoa. Yet, it is seldom done, often exploring too few cells and using basic, not highly discriminative methods. In the ejaculates of fertile boars acting as sires for artificial insemination (AI) programmes, the most frequently observed sperm abnormalities are the so-called “immature spermatozoa”, i.e. spermatozoa holding proximally-located cytoplasmic droplets. As well, spermatozoa with bent or folded tails are also commonly seen, although several other aberrant types can be seen at low frequency [2]. Here we present two cases of a previously unreported sperm defect appearing in boar studs in Finland. Both affected animals (Boars A and B) were of the Hampshire breed. Spermatozoa showed small particles scattered on their surface with a prevalence decreasing with the age of the boar. The defect in boar A was observed by a technician at an AI station when boar A was 8 months old. The number of affected cells decreased with increasing boar age and the prevalence decreased to a low level when the boar was approximately 16 months old. The boar was used only for heterospermic inseminations (i.e. insemination doses consisted of the pooled semen of several boars) and thus no fertility data of the boar was available. At the time when the technician of the AI station sent a sperm sample to the laboratory at the University of Helsinki, the defective spermatozoa had almost disappeared and only 1% of the spermatozoa were affected. Boar B was a breeding boar for on-farm inseminations. The defect was observed when the semen from a group of three boars was sent for routine quality control to the laboratory at the University of Helsinki, when the boar was approximately 11 months old at the time. The boar was used for homospermic inseminations. The fertility and litter size of the semen doses of this boar were reported comparable to other boars used on this farm (approximately 10% repeats per oestrus and an average litter size was 12.1 live piglets per farrowing).

Boar B was transferred to the clinic at the University of Helsinki and semen was collected for further studies. When the boar was slaughtered, cauda epididymal spermatozoa and fluid as well as fluids from the prostate gland, bulbo-urethral gland and the seminal vesicle were separately collected. Contents from the cauda epididymis were retrieved by pipette after cutting the cauda with a scalpel blade avoiding blood contamination. Cauda epididymal spermatozoa were extended in BTS extender (IMV Technologies) to a final concentration of 55 × 10^6^/mL, from which sperm smears were prepared.

The sperm smears were stained with eosin-nigrosin staining (Sperm VitalStain, Nidacon, Mölndal, Sweden). Two hundred cells were counted per smear and evaluated under a bright field light microscope at 1000× magnification using an oil immersion objective and classified as viable without particles (L−), viable with particles (L+), dead without particles (D−) and dead with particles (D+).

Aliquots of epididymal cauda spermatozoa were also fixed in buffered formaldehyde and transferred to the Swedish University of Agricultural Sciences, Uppsala, Sweden, where 200 spermatozoa were assessed per sample with phase contrast microscopy at 400× magnification and classified as cells with or without particles. Moreover, cauda epididymal spermatozoa were incubated in prostatic, bulbo-urethral and seminal vesicle fluids (200 µL fluid was added to 800 µL extender sperm) for 15 min at + 37 °C and the appearance of cell surface-bound particles was assessed with phase contrast microscopy as described above.

Spermatozoa were labelled with LIVE/DEAD^®^ Fixable Red Dead Cell Stain Kit (L23102, Invitrogen). Fifty microliter DMSO was added to one vial of fluorescent dye to make a stock solution. Spermatozoa were suspended in 1 mL PBS at approximately 1 × 10^6^/mL. One microliter of fluorescent dye was added to the suspension. After 30 min incubation at room temperature, spermatozoa were washed and re-suspended in 1 mL PBS twice and subsequently analysed on a BioRad MRC 1024 confocal laser scanning microscope.

Cells were fixed in a solution of 2.5% glutaraldehyde in PBS (pH 7.2) for 2 h at 4 °C. After washing, the samples were post-fixed with 1% OsO_4_ and 0.5% K-ferrocyanide in PBS for 2 h, dehydrated in graded series of acetone, and embedded in Spurr’s resin. Semi-thin sections were stained by 0.5% toluidine blue (pH 8.5) from where areas of interest were trimmed out for further ultra-sectioning. Ultrathin sections were cut by an RMC MT-7 ultramicrotome, stained with 2% uranyl acetate and lead citrate and examined on a Philips CM10 electron microscope.

**Fig. 1:**
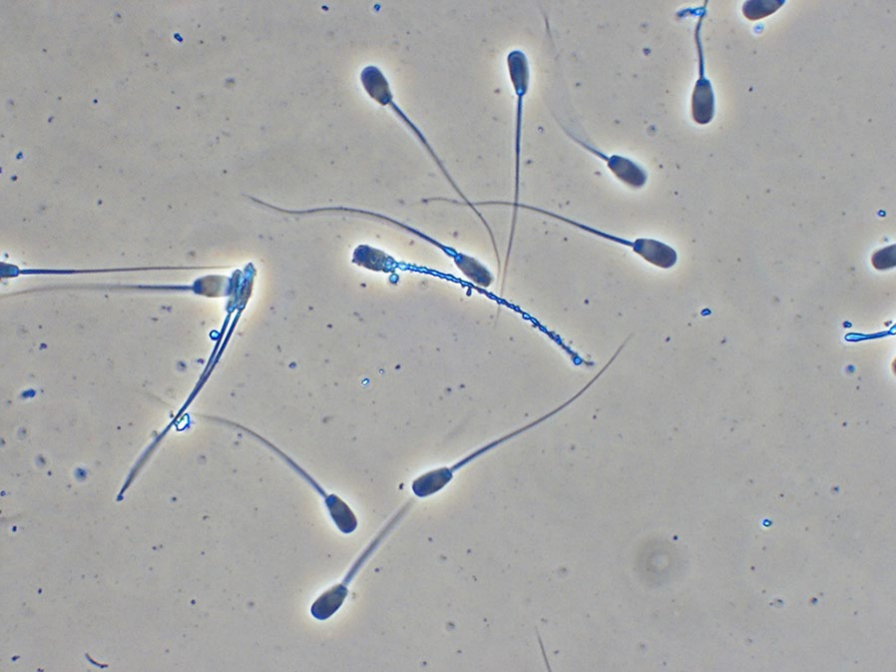
A BTS extended semen sample—one cell is covered with particles over the whole cell surface. Phase contrast microscopy

**Fig. 2:**
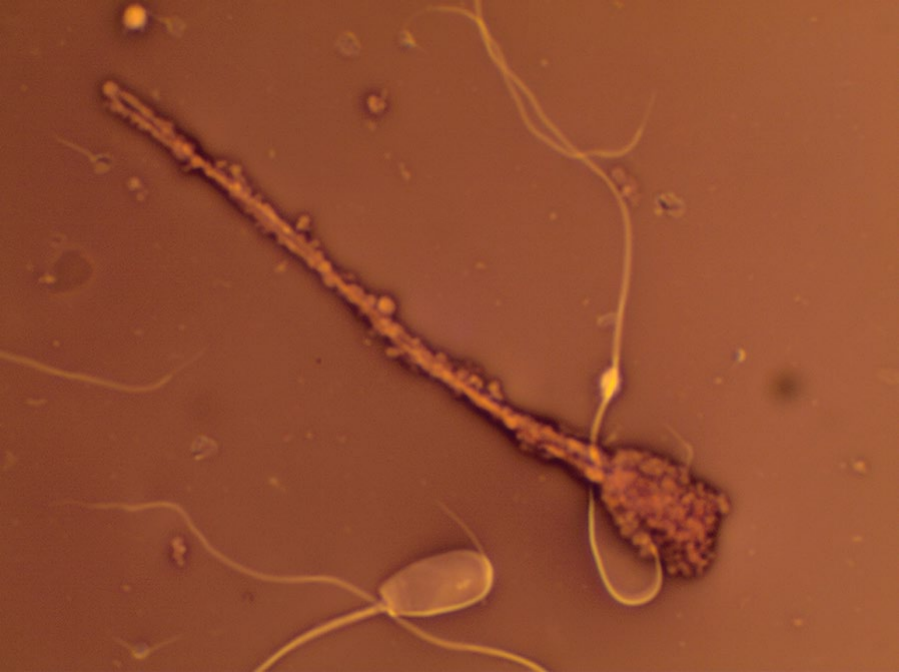
Viable and dead spermatozoa—only dead cells are covered with membranous particles. Eosin-nigrosin staining

Both eosin-nigrosin stained and formaldehyde-fixed phase contrast samples contained spermatozoa with particles attached to their surface. Moreover, eosin-nigrosin revealed that practically only dead cells had particles attached (Table 1; Figs. 1 and 2). Spermatozoa retrieved from cauda epididymis after slaughter did not contain any particles. Still at 13 months of age the prevalence of defected spermatozoa was nearly 40% in boar B.

**Table 1:** Light microscopic counting of spermatozoa of boar B without (−) or with (+) particles

Sample no.	Eosin-nigrosin %				Formaldehyde fixed %	
	L−	L+	D−	D+	−	+
1	46	3	3	48	53.5	46.5
2	70.5	2	5	22.5	60.5	39.5

Cells were further classified as viable (L) or dead (D) with eosin-nigrosin staining. The ages of the boar were 12.5 and 13 months respectively, at sampling of the two ejaculates

**Fig. 3:**
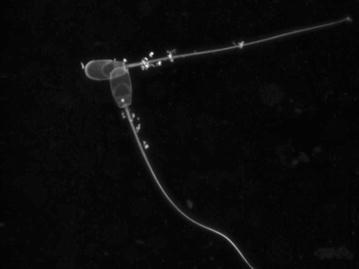
Spermatozoa with loosely attached particles after the washing steps of the fluorescent labelling with LIVE/DEAD Fixable red probe. Confocal laser scanning microscopy

Confocal laser scanning microscopy indicated that the particles were made of phospholipid membranes, and they seemed loosely attached to the cell surface as subsequent washing steps of the fluorescent labelling resulted in their detachment from the cellular surface (Fig. 3).

**Fig. 4:**
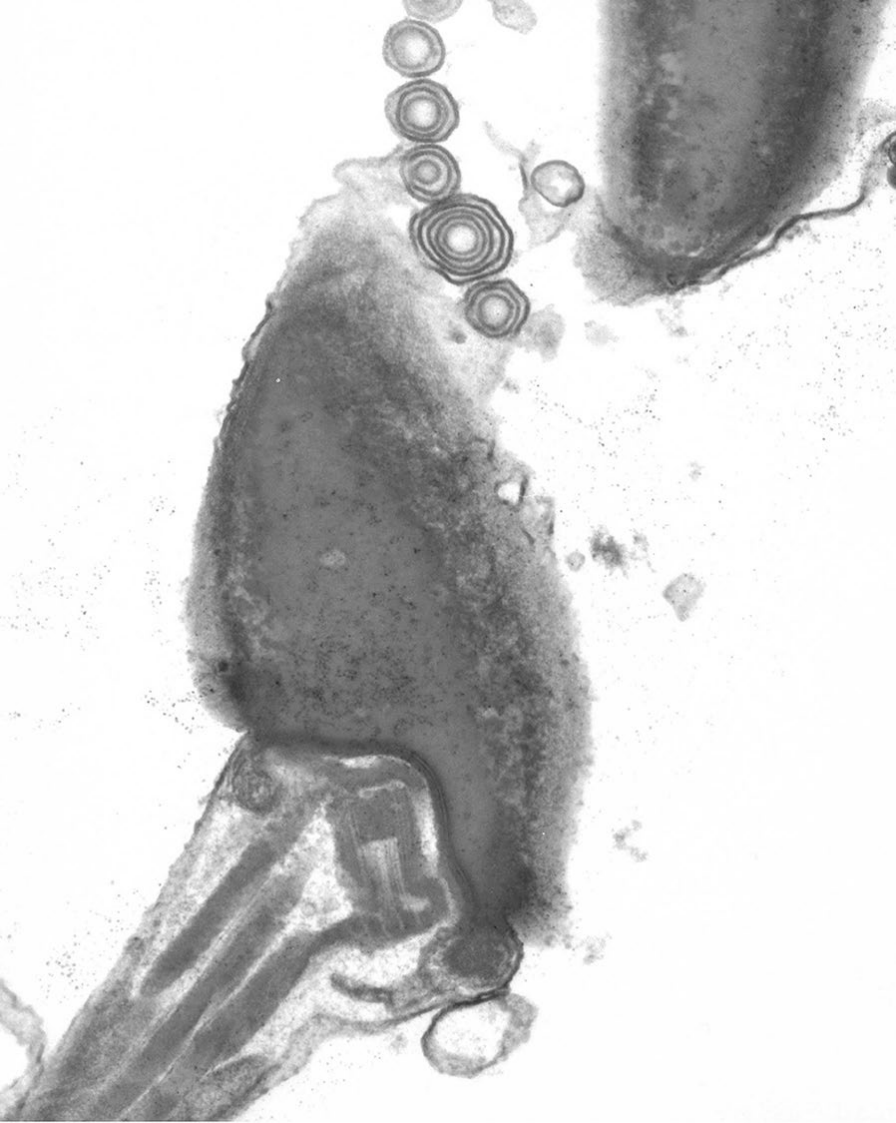
Multi-lamellar vesicles over a sperm head. Transmission electron microscopy

**Fig. 5:**
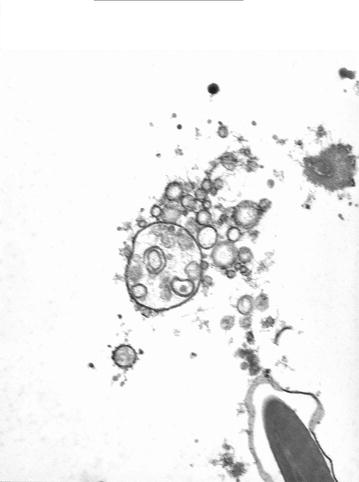
Multi-vesicular vesicles. Transmission electron microscopy

Transmission electron microscopy revealed the membrane-associated particles were multi-vesicular and multi-lamellar vesicles (Figs. 4 and 5).

Since the prevalence of the defect was very low at the time of slaughter of boar B, epididymal spermatozoa incubated with fluids from the boar genital tract did not result in a high percentage of spermatozoa with attached particles. However, we observed a few spermatozoa with such particle attachment, but only in samples incubated with seminal vesicle fluid. Incubation in fluids of the prostatic or bulbo-urethral glands did not result in sperm-particle attachment.

The presence and possible functions of multi-lamellar and multi-vesicular bodies in the semen of several mammalian species are known—as liposomes or exosomes, and classified according to their origin as epididymosomes when present in the epididymal fluid or prostasomes when present in the prostate secretion [3]. The origin and function of exosomes differ between species with intravaginal or intrauterine semen deposition [4, 5]. Epididymosomes have a role in semen maturation, while prostasomes are responsible for post-ejaculatory membrane changes [6]. Since in our case epididymal sperm did not contain unusual particles we initially classified the vesicles as prostasomes. Prostasomes can be both multi-lamellar and multi-vesicular and can be produced in other reproductive glands, too [7], although whether they can be produced by the seminal vesicles, for instance or the bulbourethral glands remains unknown. These vesicles are present in the sperm-rich fraction of the pig ejaculate and they are acrosome reaction-inducers [8]. In stallion semen (another species with a large semen volume and intrauterine semen deposition), Aalberts et al. [9] found that prostasomes bind differently to viable and dead spermatozoa; the viable cells bound vesicles only if they were capacitated and binding was restricted to the acrosome region, while dead cells show diffuse binding over the whole cell surface. The large ejaculate can carry the prostasomes into the uterus where they can bind to capacitated, viable cells. However, in our case prostasomes were ruled out since the co-incubation of epididymal spermatozoa with prostatic fluid did not result in vesicle binding to the sperm surfaces. Such binding was, on the other hand, observed after incubation in seminal vesicle fluid only. Again, species specific differences have to be taken into consideration—for example in human, the seminal vesicles do not seem to produce vesicles [10]; in bovine, most prostasome-like vesicles actually originate from the seminal vesicles [11] and are called vesiculosomes [12].

Although we were not able to determine the exact cause of the abnormal binding of vesiculosomes to dead boar spermatozoa, we classify the case as a post-epididymal multivesicular sperm defect with a favorable prognosis, since the prevalence of the abnormality decreased rapidly over age and based on the reports of the breeder the boar had average fertility and litter sizes. This unusual abnormality can be classified as “compensable” [13], i.e. when enough numbers of morphologically normal spermatozoa are present in the insemination dose, relatively high percentage of affected cells can be present without noticeable effect on fertility.
